# Man and His Enemies

**Published:** 1889-04-20

**Authors:** 


### The Doctor's Pulpit.

### MAN AND HIS ENEMIES.

### A Youth's Catechism of Health.

Our forefathers were very definite in their beliefs. They had as much faith in systematic teaching as the iron-moulder has in his methods of work. He knows by habitual experience that if he fills his box with sand, and then firmly impresses it with any model he wishes to reproduce, he will infallibly succeed in reproducing it merely by pouring in his metal when it is hot and fluid, and then waiting until it cools. Is there any analogy between youth and hot fluid metal, or between modelling-boxes and catechisms? There is no identity; that is at once admitted. But that there is an undoubted analogy worthy to be thought about and remembered is undeniable. All kinds of instruction, to be effectual, must be definite. They must be put as convincingly and stated as precisely as the proposition that two and three make five. If it be argued that many subjects of instruction and speculation are not definitely known, and that therefore they cannot be taught with such absolute precision as arithmetic, the answer is obvious. Certain elementary facts are known with the most demonstrable certainty. These, at any rate, can be taught with definiteness and authority. They are usually the most important and fundamental facts, and they should always be precisely formulated and forced home upon the intellect, the memory, and the conscience in every available way.

Physiology, including the doctrines of health, is a vague science to the outsider. Even to the half initiated it is grievously lacking in positiveness and tangibility. Many a medical man will admit, with a shrug of the shoulders, that of scientific physiology he knows little or nothing as he ought to know it. But if a knife-grinder who tramps the country, earning no more than five shillings a day, requires to be familiar with the nature and use of his grinding machine, it surely needs no demonstration to show that every grown-up boy and young man should know something of the nature and use of the parts and of the whole of his own body, which is even more fundamentally necessary to his existence and happiness than is the machine of the knife-grinder to him. That this self-evident proposition rouses about as much enthusiastic faith in the ordinary teacher of youth, and no more, than the statement that the horse gnarls his grass whilst the cow seizes a large mouthful at each bite, is a marvel which no man of science can by any possibility understand.

What makes one thousand Englishmen beat ten thousand Zulus or Soudanese in war? Knowledge of the art of war, previous preparation and discipline. What makes living generals accomplish more with twenty thousand men in a long campaign than the generals of Wellington's time could accomplish with forty thousand? Still, knowledge; knowledge of what men can and what they cannot do, of what they can and what they cannot endure; knowledge of the methods of preserving and increasing their strength; knowledge of the treatment and restoration of the wounded to health; knowledge of the nature of the machine to be employed, and of the art of making the best use of it; in short, knowledge of the physiology of an army. But is not each growing boy an army of organs in himself, and is he not setting out upon a campaign which shall be lifelong, and fraught with consequences so momentous—at any rate to himself—that no language can be found which will adequately set them forth? Is any man so insane as to despise the individual and his life campaign? He who despises the individual despises himself, despises the race, despises God who made—first the individual, and then the race. The individual *is* the race to all intents and purposes.

Every boy in his last year of school is approaching the period of his separate and independent life. He has not, up to the present time, very much needed a catechism of health, because he has been guided and controlled and subjected to the will of others. Now he is going to follow the bent of his own will. Does he not require some definite principles to guide him? Where shall he find them? He ought to find them in his memory, in his judgment, in his conscience. But how can he find them there if they have never been put there? The mind of a senior schoolboy is partly what it is by nature, but still more what it is by teaching and training. Can anyone be found so foolish as to say that such a boy is not approaching a dangerous period of life? If the parent and the religious teacher look forward with apprehension because they see him about to enter a territory which swarms with enemies to his morals, the earnest physiologist or medical man has at least an equal anxiety as he contemplates the enemies that lie in wait to destroy health and life. St. Paul, addressing his converts, strangely anticipated the feelings and the mental attitude of modern physiologists. "Your bodies," said he, "are the temples of the Holy Ghost." The physician, who is also a master in physiology, learns to look upon the body, with its admirable parts, as a kind of pure and perfect living organism, which it is desecration to poison with base contamination or to injure with careless stupidity. He reverences the human body as the sum and highest perfection of all the art and practical skill he has seen or known.

The constructors of the Church Catechism were very wise persons. Any man can see so much as that without committing himself to the principles of dogmatic theology. They prepared, not an academico-scientific body of divinity for all sorts and conditions of children—which would have been ridiculous—but a brief outline of the plain and elementary duties of just and right living, framed on a religious basis. Boys and girls with the principles of the Church Catechism in their memories, and approved by their judgment, are admirably armed from within for the conflict of life. Certain animals, it is well known, do not fight when attacked. They simply lie still and present to the enemy the defensive armour which is a part of their natural constitution—armour which has commenced with the birth and grown with their growth. These animals are perhaps more frequently victorious than those who enter upon more savage contests. It is thus that senior schoolboys should be armed from within for that part of life's battle which lies immediately ahead. They should know and be prepared for the enemies that lie in wait, and the dangers that lurk beneath the fairest outside shows. It is impossible to believe that so many young men would make shipwreck of health and of all their fairest prospects, if they were made clearly to understand the terrible nature of the disasters which result from certain ways of life that are all too common in these times.

Of course everybody feels that it is a hateful thing to make boys acquainted with all kinds of foul ways of living, with which, if left to themselves, the better among them would never become familiar. But there is no necessity for that. The elementary principles of healthy physiological life are few and simple. It is these that should be put into a form as practical and as easy to understand and remember as the Church Catechism. That is the very opposite of what is done at many schools where physiology is professedly taught. At those institutions it is taught as a science; as an academic exercise; as something to be crammed for examination purposes. Huxley has much to answer for in this regard. His "Physiology for Schools" is excellent as an elementary scientific textbook; but for all purposes of practical health improvement it is absolutely useless.

No doubt Huxley would argue that if a boy is to learn a science at all, he should learn it well. Most true; but we do not try to make every ploughboy a scientific farmer. If we did, and never taught him to plough or harrow, or sow or reap, the country would soon "go to the dogs." We teach him what he wants for immediately practical purposes; and if he displays special aptitudes, he can then pass to higher branches of study. Similarly, we should teach all senior boys the broad, fundamental principles of sound health and the art of managing both body and mind, so as to get the best possible service out of both. There is much territory to be conquered, and many difficulties to be overcome, before the system can be established which shall send every young man forward into life thoroughly equipped by knowledge and training for successful conflict with the deadly enemies which await his approach. But as scientific men add to their knowledge conscience, and to conscience apostolic fervour, so shall we advance with steady step towards a period of deeper and more practical training for the young, and therefore of greater health and happiness for manhood and for old age.

